# Development of a functional and disinfectable sewage vent high-efficiency particulate air filter system and its validation using vaporized hydrogen peroxide

**DOI:** 10.1128/aem.01269-26

**Published:** 2026-09-09

**Authors:** Jan Schinköthe, Hendrik A. Scheinemann, Sandra Diederich, Mario Lorenz, Rene Stenzel, Holger Freese, Michael Eschbaumer, Jens P. Teifke, Sven Reiche

**Affiliations:** 1 Department of Experimental Animal Facilities and Biorisk Management, Friedrich-Loeffler-Institut 39023 , Greifswald-Insel Riems, Germany; 2 Institute of Novel and Emerging Infectious Diseases, Friedrich-Loeffler-Institut 39023 , Greifswald-Insel Riems, Germany; 3 Technical Department, Friedrich-Loeffler-Institut 39023 , Greifswald-Insel Riems, Germany; 4 Institute of Diagnostic Virology, Friedrich-Loeffler-Institut 39023 , Greifswald-Insel Riems, Germany; Centers for Disease Control and Prevention, Atlanta, Georgia, USA

**Keywords:** airborne disinfection, sewage vent system, HEPA filter, hydrogen peroxide, *Vesiculovirus* (VSV), murine norovirus (MNV), *Mycobacterium senegalense*, *Geobacillus stearothermophilus*, *Bacillus subtilis*

## Abstract

Airborne disinfection of high-containment facilities is mandatory for safe operations. However, sewage vent air—particularly in large-animal experimental units—can represent a critical gap and is typically not considered in the design of high-efficiency particulate air (HEPA) filter systems. Here, we present an engineering design enabling HEPA filtration of sewage vent air from primary biological containments, including large-animal facilities and necropsy suites. We additionally evaluated vaporized hydrogen peroxide (vH_2_O_2_) fumigation as an airborne disinfection approach. Disinfection efficacy was determined by quantitative carrier testing using commercial spore carriers (CSC) coated with 10^6^ spores of *Geobacillus stearothermophilus* and compared with germ carriers coated with surrogate viruses, mycobacteria, and other spores. Temperature, relative humidity (RH), and vH_2_O_2_ concentration were monitored in real time to define process parameters and validate protocols targeting ≥4 log_10_ reduction. Inactivation efficacy differed substantially among challenge agents: CSC were least resistant to inactivation, whereas murine norovirus (MNV) on germ carriers was most challenging to inactivate. At medium-high vH_2_O_2_ concentrations (up to 520 parts per million [ppm]), increasing RH was the dominant driver of inactivation. Based on these data, we developed a biphasic disinfection cycle maintaining a saturated humidity environment with ~35% H_2_O_2_ for ≥10 h, achieving ≥4 log_10_ reduction of MNV infectivity and *G. stearothermophilus* spore viability on germ carriers. Overall, vH_2_O_2_ under high-to-saturated RH is an effective means to disinfect technical infrastructure in biological containment settings handling high-consequence pathogens.

**IMPORTANCE:**

Vent air from sewage systems in biological containment facilities where large animals are kept should be considered a contaminated waste stream. Therefore, effective high-efficiency particulate air (HEPA) filter systems that prevent mold or other moisture-related deterioration of the filters need to be installed. Airborne vaporized hydrogen peroxide (vH_2_O_2_) is an appropriate method to decontaminate these complex HEPA filter systems. High to saturated humidity is required for virucidal and sporicidal efficacy under medium to high concentration of vH_2_O_2_. Commercially available spore carriers are artificial in design, resulting in lower resistance to inactivation compared to custom germ carriers coated with non-enveloped viruses and thus should be avoided as read-out (validation means) by the biosafety community working with high-consequence pathogens in primary containments.

## INTRODUCTION

High-containment facilities include specialized units in which large animal species are housed and handled within primary containment under Animal Biosafety Level 3 Agriculture (ABSL-3Ag) or Animal Biosafety Level 4 Agriculture (ABSL-4Ag) conditions (1). These facilities comprise dedicated animal housing areas and necropsy suites designed for work with high-consequence pathogens (1, 2). As in other high-containment laboratories, their safety concept is based on layered engineering and procedural controls, including controlled access, directional airflow, pressure cascades, high-efficiency particulate air (HEPA)-filtered exhaust air, validated decontamination, and treatment of contaminated waste (1, 3). In large-animal containment, these requirements are amplified by high biomass, large effluent volumes, contaminated bedding and carcass material, and extensive wastewater interfaces, especially since the room itself is the primary containment, unlike work with small rodent species (e.g., mice, rats), which can be handled in isolated caging systems (1, 3). These features create potential release pathways that extend beyond conventional room-air exhaust and require dedicated risk assessment and engineering controls (2). In addition, regular maintenance periods of the facility with recertification of critical technical infrastructure are often required (1, 3, 4). During the preparatory phase, the shutdown, all infrastructure must be decontaminated prior to opening the facility for access by maintenance crews (5). Therefore, it is imperative to have validated disinfection protocols in place to inactivate surfaces and materials. Among them, fumigation with vaporized hydrogen peroxide (vH_2_O_2_) (6–8) has largely replaced formaldehyde (9, 10), and new methods, such as aerosolization of peroxyacetic acid (5, 11–13), have also been introduced.

Besides, disinfection of whole rooms, ducts, and equipment, HEPA filters in heating, ventilation, and air-conditioning (HVAC) systems are critical components that are contaminated with airborne pathogens, especially in large animal experimentation where the room itself is the primary containment (1–3). In addition, microorganisms on filter material can survive for up to 210 days (14). Furthermore, HEPA filters have to be recertified periodically to confirm filter integrity and efficiency (3, 4). Given that HEPA filters require annual testing and certification, and that HEPA filter housings should be designed to permit *in situ* decontamination and validation prior to filter removal, effective validated disinfection procedures are necessary to ensure safe performance testing, maintenance, and handling of potentially contaminated filter units (15, 16).

Surface disinfection with vH_2_O_2_ is a well-developed, widely established, and validated procedure (17), but the simultaneous disinfection of supply and exhaust HEPA filter systems in one setup is challenging (18). One reason is the complex architecture of the HEPA filter material itself, which absorbs excessive amounts of chemical fumigants and can reduce the concentration of disinfectant acting on the stainless-steel surfaces of the filter housings (19). Airborne disinfection is the primary method to inactivate HEPA filters, and vH_2_O_2_ is routinely applied (16, 20).

Under routine conditions, effluent from BSL 3/4 laboratories is not contaminated since infectious agents are only handled in the primary containment of a biosafety cabinet. In experimental animal facilities, especially those working with large animals, however, the animal room or necropsy suite becomes the primary containment (1–3). Consequently, sewage from these facilities must be considered contaminated with high-consequence pathogens, e.g., Ebola virus, foot-and-mouth disease virus, mycobacteria, or spores of *Bacillus anthracis* (3). Downstream, sewage undergoes thermal sterilization in the effluent disinfection system, but vent air with infectious and soil-laden droplets is generated in the piping. While HEPA filtration for air vented from sewage systems in high containment facilities handling larger animals is generally recommended (1, 2), no detailed description of a suitable drain-waste-vent air filtration system nor validated disinfection protocols for this kind of technical infrastructure are currently available.

We here propose a unique design of a sewage vent HEPA-filter system with sustained unidirectional airflow, which prevents mold and filter material disintegration. We further address the difficulties of decontaminating such a complex system and perform thorough validation of airborne decontamination processes and employ selected surrogates representing relevant resistance categories. We therefore used a published and validated panel covering bacterial spores, mycobacteria, non-enveloped viruses, and enveloped viruses (5, 21–24). We further show once more that commercial spore carriers show limitations (5, 18) in measuring disinfection efficacy for some applications.

## RESULTS

### The initial sewage vent air filter design did not pass performance qualification testing

As part of the present study, an initial sewage vent air filter configuration was subjected to performance qualification testing. During these tests, several design limitations became apparent (Fig. 1A). Although the filter housing and filter elements were selected according to the requirements of the version of the WHO Laboratory Biosafety Manual (3rd edition, 2005) valid at the time of planning, the absence of active airflow management resulted in uncontrollable humidity conditions within the filter unit. Consequently, the HEPA-filter material became wet, and the filter seal and frame were partially destroyed (see Fig. 1B). We also observed microbial and fungal growth on the HEPA-filter material (Fig. 1C). This was not acceptable, and we decided to design a new sewage vent air filter system incorporating air purging to eliminate the flaws in the initial design (Fig. 1D).

**Fig 1:**
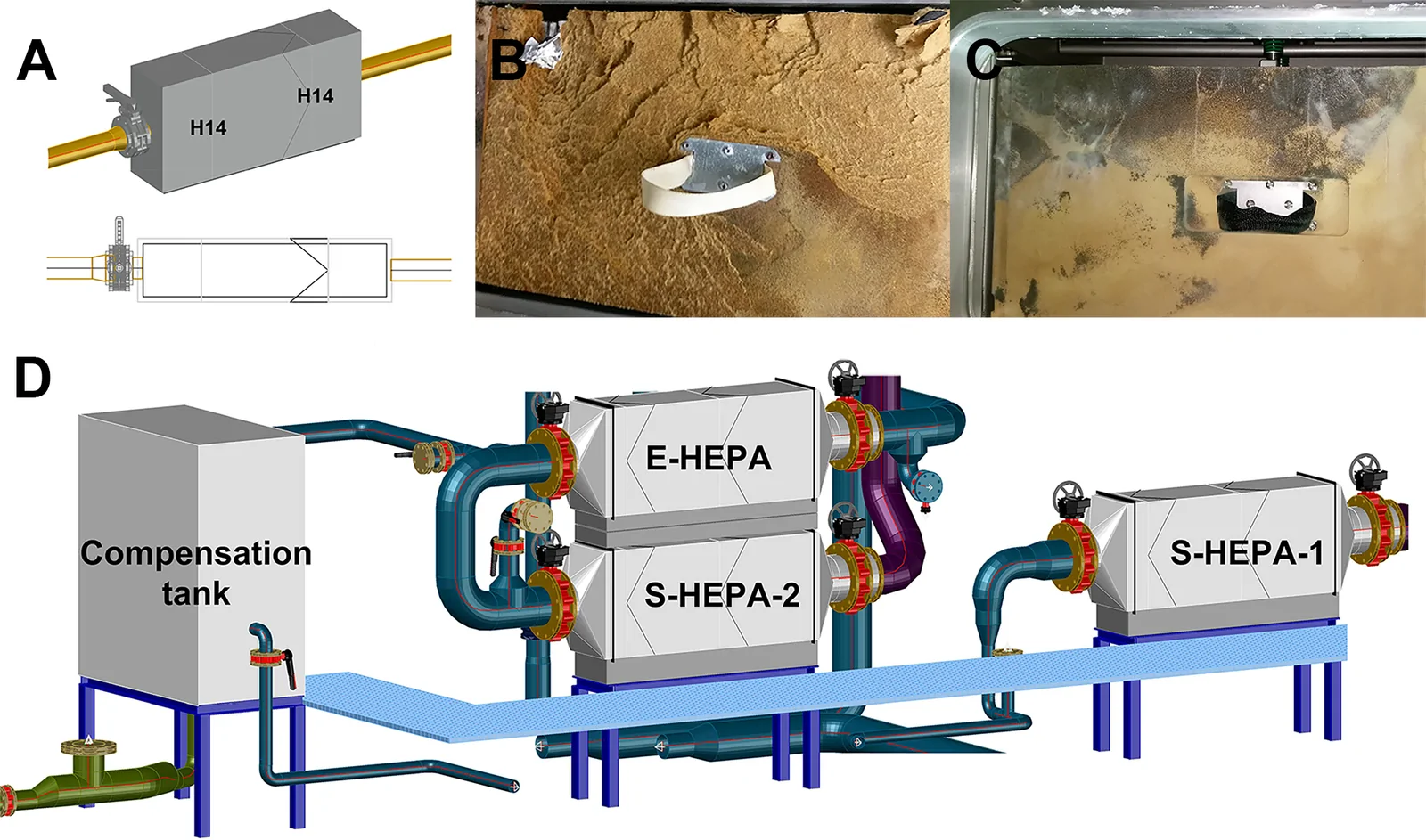
(**A**) Three-dimensional schematic of the original sewage vent air filter housing equipped with two H14 HEPA filters. (**B** and **C**) Representative pictures of a partially destroyed HEPA filter frame and the mold formation on its surface. (**D**) Three-dimensional schematic of the newly designed sewage vent air filter system, which consisted of two supply filter housings (S-HEPA-1 and S-HEPA-2), one exhaust filter housing (E-HEPA), one compensation tank, and the interconnecting ducts with dampers.

### The redesigned sewage vent air filter system is a challenge for airborne disinfection of microorganisms

Next, we developed a disinfection protocol by applying 35% H_2_O_2_ as a fumigant in a closed-loop cycle to inactivate different microorganisms on stainless-steel carriers. Before starting the disinfection cycle, germ carriers (GCs) were placed at 24 locations in the filter system (Fig. 2A). The H_2_O_2_ gas generator was placed in a closed loop, aiming to decontaminate all HEPA filter housings at once by closing all dampers next to the injection and ejection ports (Fig. 2B). The cycle program consisted of a dehumidification phase (DP), a short conditioning phase (CP), a disinfection phase (DIP), a neutralization phase (NP), and an aeration phase (AP). The complete disinfection cycle took 16.5 h. At five indicated locations, relative humidity (RH) and T were monitored in real time, with high to saturated RH values measured in the DIP (Fig. 2C). Temperature of vH_2_O_2_ peaked at up to 49.4°C in the supply duct adjacent to GC21, causing measurable heating of the ductwork, and then declined rapidly upon entering the compensation tank (19.6°C, Fig. S1A). During the disinfection cycle, up to 520 parts per million (ppm) of H_2_O_2_ as the maximum concentration at the end of DIP were measured (Fig. 2D). Complete inactivation was achieved for all commercial spore carriers (CSC) (6 log_10_ reduction) and for almost all GCs coated with vesicular stomatitis virus (VSV) (6.52–6.74 log_10_ reduction) (Fig. 2E and Table S1). For all GCs coated with *Mycobacterium senegalense*, a reduction greater than 4 log_10_ was achieved (5.07–6.84 log_10_). This was not the case for GCs coated with *Bacillus subtilis* or murine norovirus (MNV). Most of these GCs had been placed in the three HEPA filter housings. When we analyzed GCs 1–12 separately, stark differences became apparent, with MNV as the most tenacious and commercial spore carriers coated with *Geobacillus stearothermophilus* as the easiest to inactivate (Fig. 2F). Because the pronounced location-dependent differences in GC inactivation suggested that local environmental conditions might influence decontamination efficacy, we analyzed GC locations for which simultaneously recorded RH data were available (locations 2, 6, 10, 14, 22). The association between RH and log_10_ reduction was assessed by univariate linear regression and Spearman correlation (Fig. 2G and Fig. S1B). While no effect was seen for VSV (*r*² = 0.00, *P* = 0.82) and CSC, a significant association between RH and reduction of infectivity was evident for MNV (*r*² = 0.63, *P* = 0.00), MS (*r*² = 0.50, *P* = 0.00), and BS (*r*² = 0.31, *P* = 0.03). Similar to the regression models, the correlation between log_10_ reduction and RH was strongest for MS (*r* = 0.77, *P* = 0.00) and MNV (*r* = 0.76, *P* = 0.00) and moderate for BS (*r* = 0.58, *P* = 0.02), while no significant correlation was seen for VSV (*r* = 0.08, *P* = 0.78) and commercial spore carriers (no significance because of zero variance).

**Fig 2:**
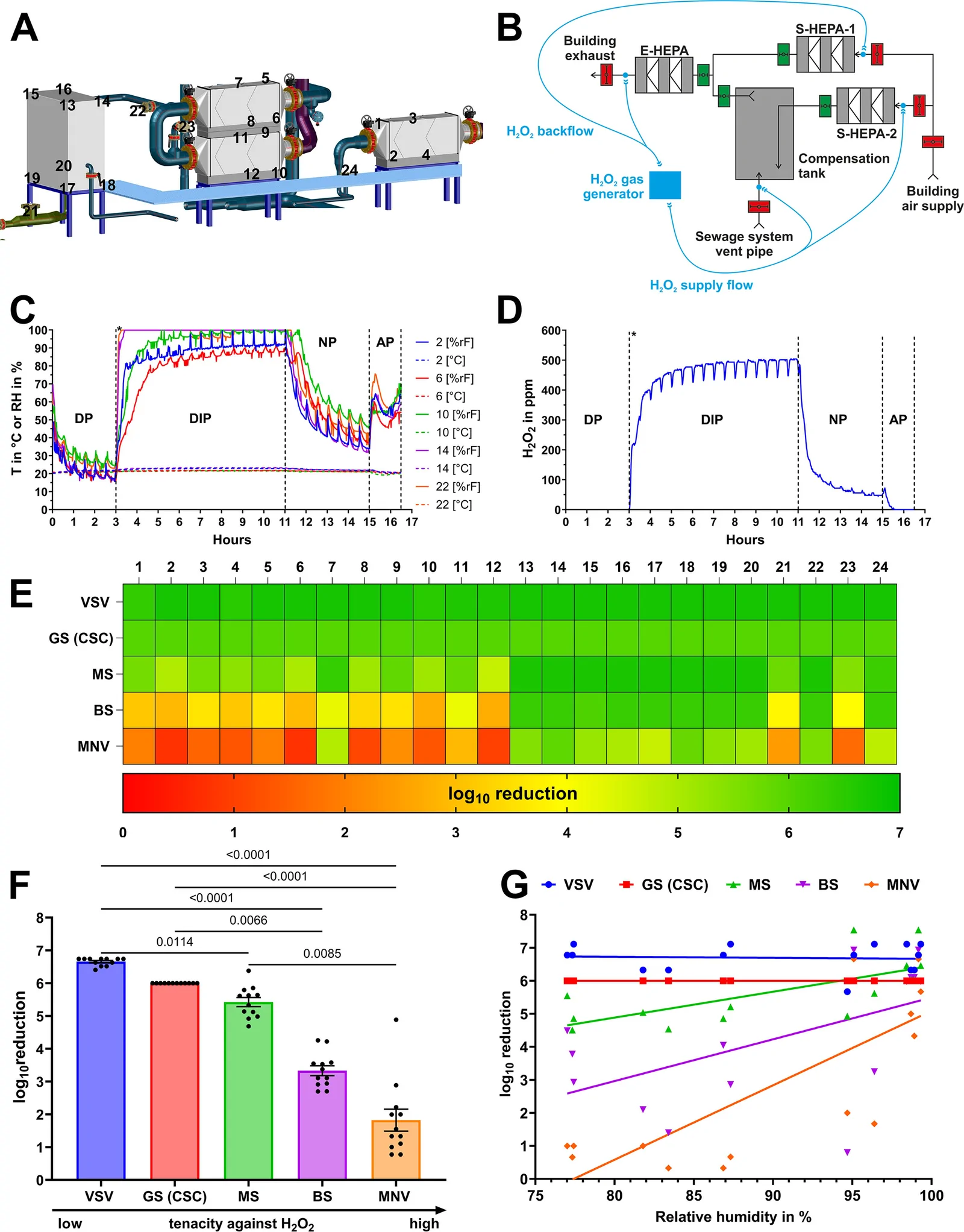
(**A**) Three-dimensional schematic of the sewage vent air filter system with locations of each germ carrier (1–24) during H_2_O_2_ disinfection. (**B**) Two-dimensional schematic of the disinfection setup showing the supply and backflow of vH_2_O_2_ and the dampers in open (green) or closed (red) position during the disinfection cycle. (**C**) Means (*n* = 3) of relative humidity in % and temperature in °C during the dehumidification phase (DP), conditioning phase (CP), disinfection phase (DIP), neutralization phase (NP), and aeration phase (AP) at relevant germ carrier locations (indicated by numbers). (**D**) Mean H_2_O_2_ concentration (*n* = 3) in parts per million (ppm) measured during the disinfection cycle. (**E**) Heat map with mean log_10_ reductions (*n* = 3) of vesicular stomatitis virus (VSV), murine norovirus (MNV), *M. senegalense* (MS), spores of *B. subtilis* (BS), and *G. stearothermophilus* (GS) as commercial spore carriers (CSC). Numbers on the y-axis indicate the location of spores. (**F**) Focused analysis of data at locations 1–12 in the three HEPA filter housings shown as mean of log_10_ reductions ± SD (*n* = 12) for each microorganism. Statistical significance was calculated with the Kruskal-Wallis test in Prism 11 with Dunn’s post hoc test for multiple comparisons. (**G**) Relationship between relative humidity and log_10_ reductions of microorganisms measured at germ carrier locations 2, 6, 10, 14, 22 (*n* = 5) derived from three disinfection runs. Each point represents a single measurement and lines depict simple linear regression.

Macroscopic analysis of the compensation tank revealed widespread condensation on interior surfaces, with partial destruction of the chemical indicators when recovered after each disinfection cycle but no apparent damage to the compensation tank itself (Fig. S1C).

### A modified disinfection cycle and constant saturated RH enable efficient disinfection of murine norovirus and spores of *G. stearothermophilus*

The insufficient reductions of MNV and spores of *B. subtilis* forced us to modify the disinfection cycle in several ways. Since the supply HEPA filter housings are not contaminated during routine operation, we excluded them from our disinfection efforts. Instead, we focused on the contaminated infrastructure: the exhaust HEPA filter, the compensation tank, and the connecting ducts. The schematic of the closed dampers and ports for distribution of the vH_2_O_2_ are given in Fig. 3A. The modified disinfection cycle consisted of a shorter dehumidification phase (DP), a longer disinfection phase (DIP), and an additional 2 h incubation phase (IP), where H_2_O_2_ was not recycled, resulting in an overall cycle length of 22.5 h (Fig. 3B). Consequently, the mean amount of vH_2_O_2_ increased by 73.40%. The mean RH values in this setup were at the saturation limit during the entire disinfection phase at locations 1, 8, and 11, respectively (99.9% ± 0% across three independent runs). During the incubation phase, RH decreased partially, most prominently at location 8, where the lowest mean RH (68.4% ± 4.8%) was measured (Fig. 3B), which most likely indicates absorption by the HEPA filter material.

**Fig 3:**
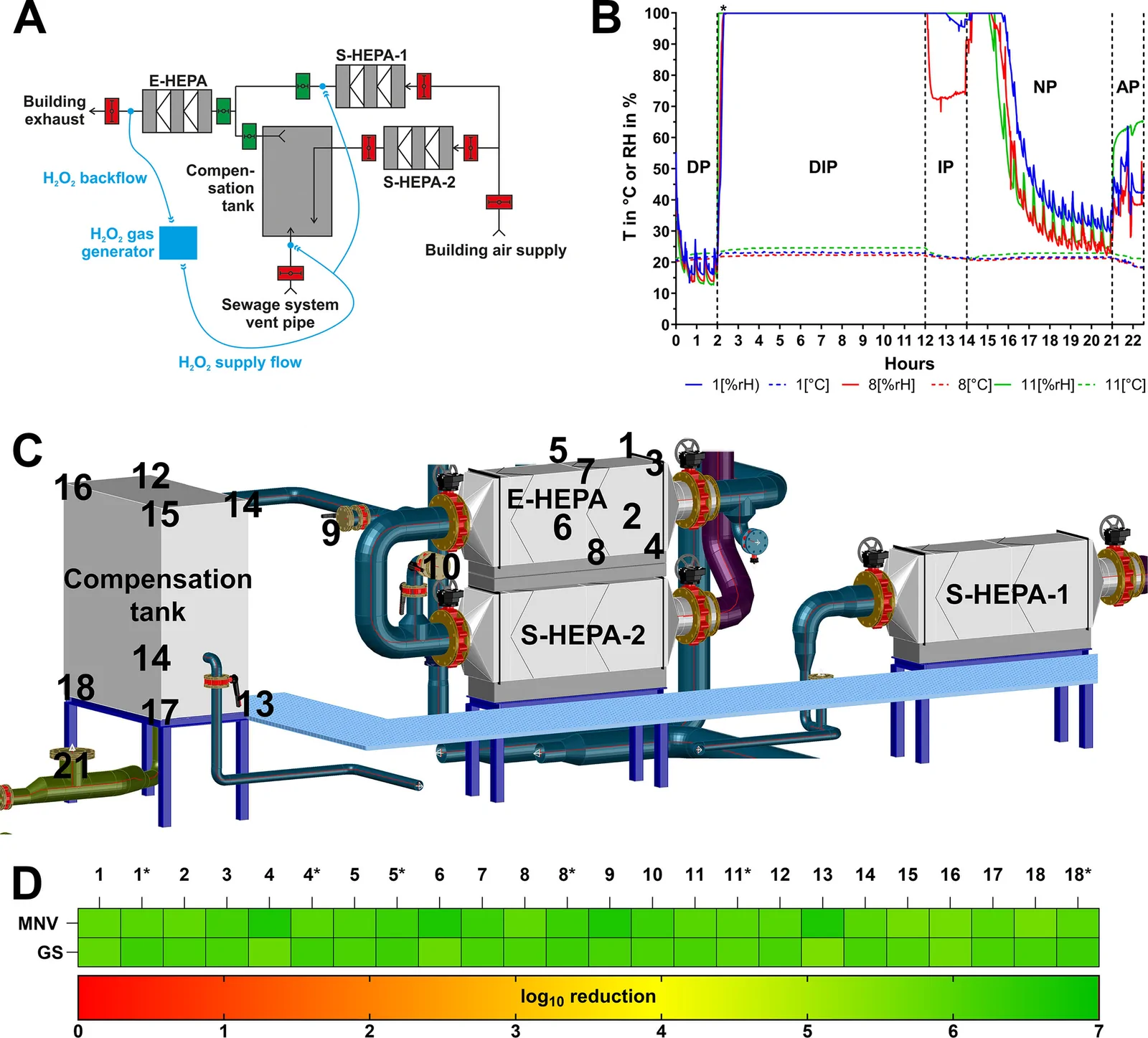
(**A**) Two-dimensional schematic of the newly designed disinfection setup showing the supply and backflow of vH_2_O_2_ and the dampers in open (green) or closed (red) position during the disinfection cycle. (**B**) Means (*n* = 3) of relative humidity in % and temperature in °C during the dehumidification phase (DP), conditioning phase (CP), disinfection phase (DIP), incubation phase (IP), neutralization phase (NP), and aeration phase (AP) at relevant germ carrier locations. (**C**) Three-dimensional schematic of the sewage vent air filter system with locations (1–18) of each germ carrier during H_2_O_2_ disinfection. (**D**) Heat map with mean log_10_ reductions (*n* = 3) of murine norovirus (MNV) and *G. stearothermophilus* spores (GS).

GCs were placed at 18 locations (Fig. 3C), and at 5 locations, additional pairs of GCs with soil load were placed to assess the influence of organic matter on disinfection efficiency. With the revised H_2_O_2_ distribution, disinfection was highly effective, yielding near-complete inactivation of all germ carriers coated with murine norovirus (MNV) (5.66–6.66 log_10_ reduction) and *G. stearothermophilus* (5.57–6.29 log_10_ reduction) (Fig. 3D and Table S2). Mean log_10_ reductions did not differ between the GC types (*P* = 0.91). Likewise, the presence of a soil load had no detectable effect on log_10_ reduction compared with no soil load (*P* = 0.29) (Fig. 3D and Table S2).

During the disinfection cycle, the RH values remained at the saturation limit. After removal of the GCs, soil-loaded carriers showed visible bubble formation/effervescence, whereas carriers without soil load were primarily covered by condensation. This observation was recorded descriptively and was not quantified (Fig. S1D). Chemical indicators showed similar findings as outlined earlier.

### The addition of a soil load preserves viability of microorganisms over time

We then assessed the time-dependent environmental stability of the microorganisms at 22°C and 30% RH for up to 28 days in the presence or absence of a soil load (Table S3). Over 28 days, both viruses declined sharply in the absence of soil load (Fig. 4A and B), reaching low endpoint titers (VSV 2.2 log_10_; MNV 2.3 log_10_). By contrast, the soil load consistently mitigated viral decay and preserved substantially higher titers at day 28 (VSV 5.0 log_10_; MNV 5.4 log_10_). For *M. senegalense* (Fig. 4C), the soil load also moderated the rate of decline early on, but this effect was minimal by day 28, with nearly identical endpoint CFU values (2.9 vs 3.0 log_10_). *B. subtilis* spores (Fig. 4D) remained essentially unchanged throughout the observation period, and viability was indistinguishable with versus without soil load (8.0 vs 8.1 log_10_).

**Fig 4:**
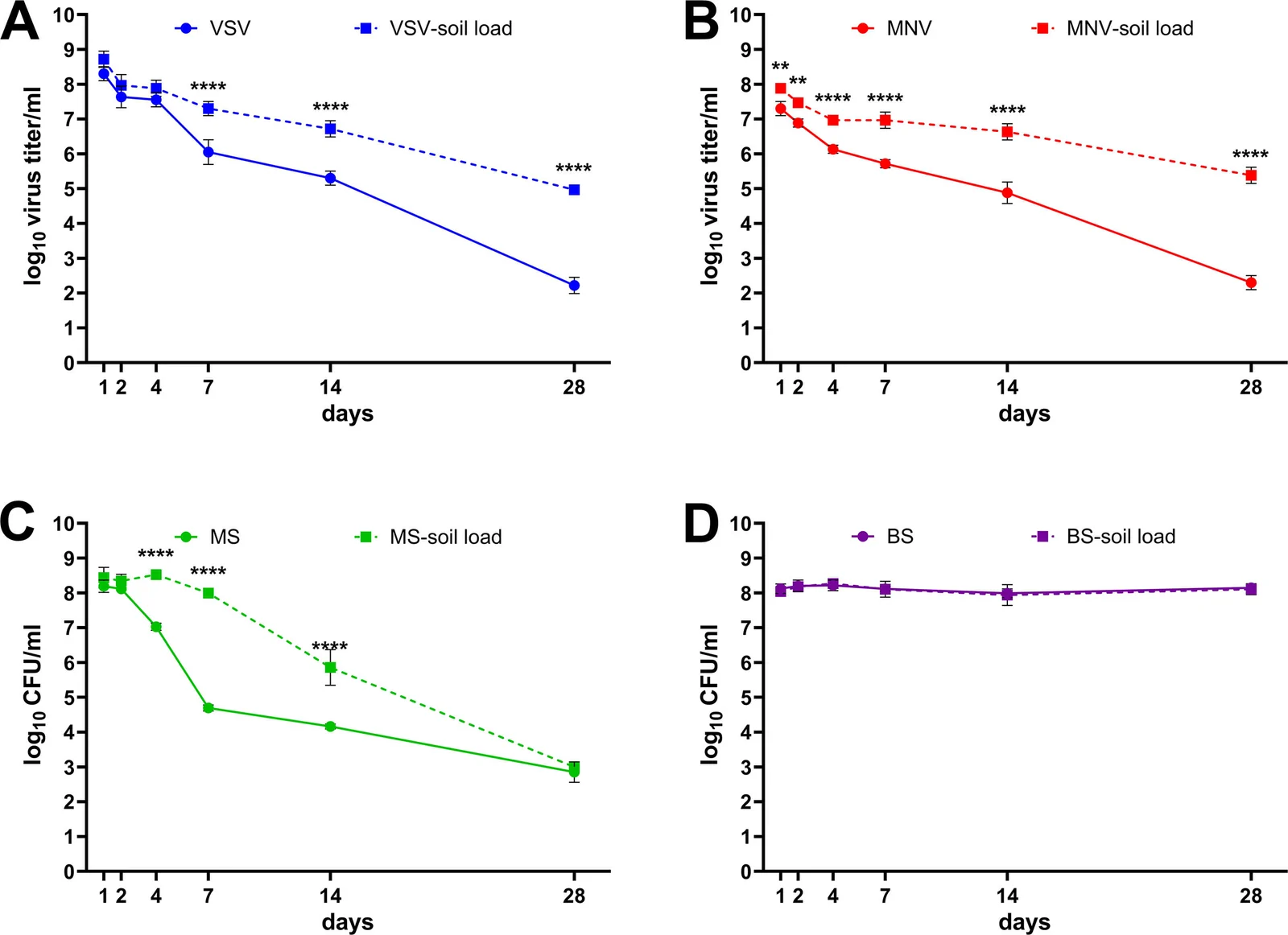
Effect of storage time and soil load on microorganism viability at 30% relative humidity and 22°C. Microorganism titers were determined after 1, 2, 4, 7, 14, and 28 days of storage in the absence or presence of a soil load. (**A**) Vesicular stomatitis virus (VSV), (**B**) murine norovirus (MNV), (**C**) *Mycobacterium senegalense* (MS), and (**D**) *Bacillus subtilis* spores (BS). Viral titers are expressed as log₁₀ virus titer/ml, whereas bacterial titers are expressed as log₁₀ CFU/mL. Each data point represents the mean ± SD of three technical replicates per time point. Differences between microorganisms stored with and without soil load were analyzed at each time point using two-way ANOVA followed by Šídák’s multiple-comparisons test. **P < 0.01; ****P < 0.0001.

## DISCUSSION

In this study, we present a complex air filtration system that enables effective filtration of potentially contaminated sewage vent air of high humidity in large-animal facilities working with high-consequence pathogens.

In the current literature, most articles describing the physical and technical infrastructure of high-containment facilities handling large animals do not explicitly mention HEPA filtration of air vented from the sewage system (1–3). As in our initial planning with one exhaust HEPA filter, it is conceivable that many designs do not adequately address the potential issues associated with high humidity of vent air. Periods of high humidity are the cause of swelling filter media and cardboard frames, which further become a suitable substrate for subsequent growth of molds (25). In our case, uncontrolled environmental conditions that allowed fungal growth partially destroyed the initially installed HEPA filters. This is an unacceptable biocontainment risk that must be addressed properly. The separation of the single HEPA housing from the main support air HVAC system by installing an additional fan that provided a permanent unilateral airflow is key in our redesigned sewage vent air filter system. The basic design enables high humidity levels to be compensated for by constantly flushing the filter cells with conditioned supply air, which prevents the formation of mold and the destruction of HEPA filters. However, the addition of more than one HEPA filter housing, significant lengths of interconnecting ductwork, and the compensation tank is a formidable challenge for standard HEPA filter disinfection protocols.

### The success of vH_2_O_2_ at medium-high concentration depends on increasing relative humidity

The disinfection of HEPA filters is a routine practice and mandatory in high-containment facilities (1, 4). Vaporized H_2_O_2_ is widely applied and has been proven a successful method. Its efficacy is demonstrated by the inactivation of commercial spore carriers (7, 25) or self-designed carriers with a variety of viral (17, 26), (myco-)bacterial (27), or spore-forming pathogens (28). To our surprise, the analysis of three independent inactivation cycles using vH_2_O_2_ of the entire sewage vent air filter system still revealed significant residual viability of *B. subtilis* spores and of MNV coated on GCs, while all commercial spore carriers were completely inactivated. These findings were of special importance to us, since *G. stearothermophilus* is accepted as the most resistant spore-forming bacillus and therefore, the gold standard in evaluating vH_2_O_2_-based disinfection methods (25, 29).

In general, disinfection success is multifactorial, and many parameters have to be considered. Especially, the concentration of vH_2_O_2_ and exposure time are important factors. Higher vH_2_O_2_ concentrations are beneficial to disinfection success (30, 31). In our first approach, the disinfection period was 8 h with a vH_2_O_2_ concentration of up to 520 ppm H_2_O_2_. Others have reported a 4–6 log_10_ reduction of spores at 300 ppm for 8 h (28). Claims of broad virucidal effects of H_2_O_2_ vapor can be found in the literature, but this is derived from small-scale experiments in pass-through boxes with up to 1,211 ppm of H_2_O_2_ for 0.5 h (17). Stuart et al. (19) state that the exposure time might influence the outcome of inactivation efficacy using vH_2_O_2_. In their experiment, vH_2_O_2_ dwelled for 30 min, whereas formaldehyde dwelled for 18 h, the latter being described as the more efficient biocide (19). However, our analysis indicates that exposure time alone cannot be the reason for the observed differences in log_10_ reductions.

The most controversial factor is relative humidity, and whether dry (vapor) or wet H_2_O_2_ application is the most effective method. Some studies praise the dry vapor approach, claiming that this form of H_2_O_2_ is more effective than liquid H_2_O_2_ (32, 33). However, most studies lack detailed measurements of RH in general and at the actual locations of the exposed GCs (19, 27, 28), which cannot rule out invisible condensation (“microcondensation”). This is of special relevance, since other authors emphasize the importance of microcondensation. Unger-Bimczok et al. showed higher water content and increased microcondensation as necessary for enhanced microbial inactivation (31). The microbicidal effect was uncoupled from the water content in air at a high H_2_O_2_ concentration of >800 ppm, concluding that saturated humidity will not enhance spore kill above this H_2_O_2_ concentration. In our setting with medium-high vH_2_O_2_ concentration, differences in log_10_ reductions for the different microorganisms used were most obvious in locations with moderate to high RH (HEPA housings), whereas saturated locations (compensation tank) showed higher log_10_ reductions in general. Linear regression and correlation analysis showed clearly that rising RH values determine the success of airborne disinfection with moderate vH_2_O_2_ concentrations, which is in agreement with the findings of Unger-Bimczok (31).

In this regard, we further want to highlight our approach to monitor environmental conditions at the location of disinfection in real time. These above-mentioned methodological differences make comparisons of studies difficult. Unfortunately, the published CEN guideline for airborne disinfection testing explicitly sets an RH range of 50%–75% as an obligatory experimental condition (34).

### Saturated humidity levels are required for >4 log_10_ reduction of germ carriers

The focus of our disinfection efforts on the contaminated exhaust HEPA filter, connecting ductwork, and the compensation tank allowed constant saturated humidity. In addition, the exclusion of four supply HEPA filters from the process removed a considerable amount of absorptive material and improved the overall outcome. However, even in the optimized protocol, 7 out of 18 (39.89%) GCs coated with MNV and 9 out of 18 (50%) coated with *G. stearothermophilus* spores showed low amounts of residual infectivity. In the final disinfection cycle, real-time monitoring of vH_2_O_2_ was not possible due to logistic problems. This is a clear disadvantage since monitoring data in ppm cannot be provided; however, since significantly more H_2_O_2_ (73.60%) was vaporized in this cycle, a concentration greater than 520 ppm can be assumed.

As outlined earlier, the complete inactivation of commercial spore carriers coated with *G. stearothermophilus* was remarkable. The claim of *G. stearothermophilus* as the most resistant spore is based on the study of Meszaros et al. (29). In their study, 10 µL of spore suspensions were manually inoculated on stainless steel coupons at a final concentration of 10^6^ CFU/coupon and air-dried at ambient temperature (29). This inoculation method resembles a liquid spot, and one can assume a multilayered spore inoculum. But this is different from currently available commercial spore carriers, which are artificial in design. There are two major differences: firstly, commercial carriers are produced at large scale and in a reproducible fashion by spraying spores evenly in a monolayer (35). We have previously verified this by scanning electron microscopy (SEM) (5). Secondly, SEM imaging revealed that the carrier material itself was extremely smoothly polished and does not resemble ordinary stainless-steel surfaces, which have crevices and niches (5). The lower inactivation efficiency of vaporized H₂O₂ observed here against non-enveloped viruses (MNV) compared to bacterial spores (Fig. 2E through G) has already been demonstrated by us elsewhere when suboptimal disinfection protocols are used. This even applies to enveloped viruses to a slightly lesser extent (36). This discrepancy was also recognized by Kümin et al. They described a ≥6 log_10_ reduction (i.e., complete inactivation) of all commercial spore carriers, while non-enveloped foot-and-mouth disease virus was not inactivated (18). Therefore, we decided to use self-prepared spore carriers of *G. stearothermophilus* and the murine norovirus with the aim to challenge our final disinfection protocol with the most tenacious spore and virus.

### Soil-loaded germ carriers were no challenge for the final disinfection protocol, but the soil load extends viability over time

In the final disinfection cycle, soil-loaded GCs were added to the experimental setup. Overall, we observed no differences in the disinfection efficacy. The literature on quantitative carrier testing with and without soil load in airborne disinfection is expanding in recent years. Some studies argue a protective effect of organic contamination, especially by blood (19, 29). We could not observe such a protective effect on the GCs with high soil load, even when sheep erythrocytes were added to the soil load. On the contrary, it has been reported that reactive oxygen species (ROS) can create reaction products with organic molecules that can themselves function as ROS (37). This has been described for BSA (38) and as a self-perpetuating peroxidation (39) of lipids, which are present in the cell membranes of sheep erythrocytes. Both factors might have influenced our experiments, as seen in the prominent reactions on germ carrier surfaces with soil load. Further experiments with different soil loads and direct measurements of ROS in the soil-loaded inocula are necessary to test this hypothesis.

Although data on the viability of microorganisms over time are scarce in the published literature, it is highly relevant for risk assessment. Therefore, we examined the viability of our viruses, mycobacteria, and spores over time, both with and without a soil load. Viruses and mycobacteria without soil load showed a greater than 4 log_10_ reduction after storage for 28 days, while soil-loaded viruses were more stable than mycobacteria. Only limited data are available for each microorganism. For instance, Riddell et al. investigated the stability of the enveloped severe acute respiratory syndrome coronavirus 2 (SARS-CoV-2) suspended in BSA and mucins to mimic salivary fluid. Similar to our findings, SARS-CoV-2 was still detectable after 28 days post-inoculation (40).

Our work on MNV is in alignment with findings from colleagues, who have shown 3.28 log_10_ of viable virus at 14 days post-inoculation at 30% RH and 90 days at 10% RH, respectively (41). They further highlighted an RH corridor around 50% in which MNV (and most likely other viruses) are most vulnerable, whereas 10% or 100% of RH preserved most of the viral particles. The spores showed no signs of reduction, which was an expected finding, since reports of spores of *Bacillus atropheus* being viable for up to 210 days exist (14).

### Conclusion

In this study, we suggest to the biosafety and engineering community an efficient setup of a sewage vent air filter system for operation under primary containment conditions. We further show that the relative humidity at medium-high vH_2_O_2_ concentration is essential for a successful disinfection of microorganisms.

Our findings replicate the limitations of commercial spore carriers, which clearly underscores the need to select appropriate and relevant test organisms or surrogates for the evaluation of the disinfection efficacy, especially in BSL-3 and BSL-4 facilities handling large animals.

## MATERIALS AND METHODS

### Initial design of sewage vent air HEPA filter housing

The initial filter housing was a passive system of standard design (Krantz, Germany), without forced ventilation or fumigation ports. It was installed in the sewage vent duct to filter contaminated air from the effluent collection system of the animal facility and the necropsy suite of the veterinary BSL-4 containment (FLI, Greifswald-Riems). It was equipped with two sequentially placed H14 HEPA filters (Camfill, Sweden, Fig. 1A).

### Development of a functional sewage vent air HEPA filter system

We found no articles in a search of Google, PubMed, CAB Direct, Web of Science, and Scopus with search terms “sewage, air, HEPA, filter, biosafety, high-containment, disinfection, hydrogen peroxide, or formaldehyde,” suggesting that no specific descriptions of this technical infrastructure or its disinfection have been published. The new design uses fans to generate a forced unidirectional airflow separate from the air stream of the main HVAC. In total, three HEPA-filter housings (Camfil, Sweden) were installed, two of them on the supply side and one for the exhaust HEPA filters (each ~6 m³ in volume, Fig. 1D). Each filter housing was equipped with two sequentially placed HEPA filters of class H14 (Camfil). The filter housings were connected by ~20 m of ductwork (polyphenylene sulfide). The three HEPA filter housings are ventilated by a fan (Mietzsch, Germany) drawing air from the building HVAC system. This special setup creates a constant unidirectional airflow and avoids high relative humidity levels within the sewage vent system, simply by allowing incoming air to expand in a compensation tank (~2 m³ in volume, Fig. 1D).

### Disinfection of the newly constructed sewage vent air HEPA filter system with vH_2_O_2_

The filter housings, ducting, and the compensation tank had to be disinfected by fumigation with vH_2_O_2_. We used a commercial gas generator (Geschko, PEA, Germany) which vaporizes and recycles 35% H_2_O_2_ in a closed cycle.

Two disinfection cycles were developed, each consisting of a dehumidification phase (DP), conditioning phase (CP), disinfection phase (DIP), neutralization phase (NP), and an aeration phase (AP). The second cycle included an additional incubation phase (IP). The cycle parameters were as follows.

Cycle 1: DP = 180 min, CP = 4 min (5.0 g H_2_O_2_/min), DIP = 476 min (3.2 g H_2_O_2_/min), NP = 240 min; total volume of vaporized H_2_O_2_ = 1,543.63 mL ± 0.21 (*n* = 3); RH and T data loggers at locations 2, 6, 10, 14, 22, and one high-concentration H_2_O_2_ sensor (POLYTRON 7000, Dräger, Germany) at the combined outlet behind the exhaust and supply HEPA filter #2 (Fig. 2B).

Cycle 2: DP = 120 min, CP = 6 min (6.0 g H_2_O_2_/min), DIP = 594 min (4.5 g H_2_O_2_/min), IP = 120 min, NP = 420 min; vaporized H_2_O_2_ = 2,676.67 mL ± 38.53. In the second cycle, both supply side HEPA filter housings were excluded from the disinfection loop (Fig. 3A), resulting in different placement of GCs and RH probes (locations 1, 8, 11).

### Testing and read-out of commercial spore carriers

Disks coated with 2.2 × 10^6^ spores of *G. stearothermophilus* (Spordex, Steris, USA) with a D-value of 0.8 min were used within their Tyvek pouches, and chemical indicators (Steraffirm, Steris, USA) were placed in tandem at different locations. After disinfection, each spore carrier was placed into a tube containing 1 mL trypticase soy broth (Carl Roth, Germany). To recover the spores from the stainless steel, the surfaces were scrubbed with a sterile pipette tip, and the tube was vortexed twice for 30 s.

An untreated disk was used as a positive control. The spores were titrated in 96-well U-bottom plates, incubated at 60°C for 7 days, and the most-probable number (MPN) method was used to quantify the success of disinfection as described before (9).

### Self-prepared germ carriers

Stainless-steel carriers (GK Formblech, Germany) were procured with an EN 10088-2 quality 2B surface treatment on both sides. They were rectangular, measuring 16 × 60 mm with a thickness between 1.2 and 1.5 mm depending on the lot. Carriers were immersed in 5% Decon 90 solution (Decon Laboratories, UK) for 60 min, then in 70% ethanol for 15 min, rinsed in demineralized water, and air-dried in a biosafety cabinet. All carriers were sterilized with dry heat at 180°C for at least 2 h. All carriers were reused multiple times until pitting of the stainless-steel surfaces became visible.

#### Germ carrier coating and recovery of microorganisms

From thawed stock solutions, 50 µL of each microorganism suspension was coated on stainless steel carriers as described elsewhere (5, 12, 42), by spreading out with a horizontally held pipette tip (10 µL per cm^2^). In experiments with a soil load, the category “high organic load” was chosen according to published guidelines (43) to represent a realistic surrogate for large-animal sewage aerosols. Briefly, to obtain 50 µL of high organic load inocula for stainless-steel surfaces, 25 µL of each microorganism suspension was mixed with 25 µL of the high organic load preparation. The high organic load consisted of bovine serum albumin (BSA) and a sheep erythrocyte suspension, with a final concentration of sheep erythrocytes and BSA in the test procedure at 3 mL/L and 3 g/L, respectively. Soil-loaded carriers were stored at controlled environmental conditions (22°C, 30% RH) for up to 28 days.

With spore and virus suspensions, a homogeneous coating was obtained, but the hydrophobic mycobacteria formed visible clots. To establish a homogeneous distribution of mycobacteria, three washes with PBS, followed by resuspension in PBS with 1% polysorbate 80 and pipetting up and down (10 times) through a 0.4 mm (27G) blunt cannula were performed. The coated germ carriers were air-dried in a biosafety cabinet. After fumigation with vH_2_O_2_, germ carriers were collected and washed with 1 mL of tryptic soy broth for spores and mycobacteria or with cell culture media for viruses, respectively. The carriers were placed upright in 12-well plates and washed ten times by manually up- and down pipetting of 750 µL of the liquid for viruses and 1,000 µL for spores and mycobacteria. Carriers with spores and mycobacteria were also scrubbed with a horizontally held pipette tip.

#### Controls

Drying controls were used in each trial and were stored in closed boxes immediately after air drying. Stainless steel carriers coated with cell culture supernatant or bacterial growth medium (1:1), respectively, served as toxicity controls. The use of disinfectant neutralizer was avoided since all stocks had titers high enough to allow the verification of a reduction of infectivity greater than 4 log_10_, which was defined as successful disinfection (44).

#### Viruses

The cells were routinely propagated in the presence of 10% fetal bovine serum, but for some viruses, media with different serum content were used as outlined elsewhere (5). Cells, media, and viruses were kindly provided by the Riemser Virusbank (RVB, FLI, Germany) or the Riemser Zellbank (CCLV-RIE, FLI, Germany). Murine norovirus (MNV, RVB 0651; strain S99) was propagated in RAW 264.7 (CCLV-RIE 0996) cells, whereas vesicular stomatitis virus (VSV, RVB 0030) was propagated in LFBK-αVβ6 (CCLV-RIE 1419) cells. Virus stocks were prepared by inoculating a >90% confluent layer of cells, followed by incubation at 37°C and 5% CO_2_ until a prominent cytopathic effect developed. Virus was harvested by freezing and thawing of cells to release viral particles. After thawing, the supernatant was centrifuged at 2,000 × *g* for 10 min and stored in aliquots at −80°C until use. To estimate viral titers, endpoint titration was performed in 96-well plates with an evaluation of the cytopathic effect after 5–6 days. Tissue culture infectious doses (TCID_50_/mL) were calculated by the Spearman-Kärber method (45). The limit of detection of infectivity was 1.8 log_10_ TCID_50_/mL. In some instances, condensation of H_2_O_2_ on the carrier surface caused non-specific cytotoxicity and raised the limit of detection to 2.8 log_10_ TCID_50_/mL.

#### Bacteria and spores

From the commercially acquired spore carriers, *G. stearothermophilus* was isolated and used to prepare stable spore stocks as described earlier (5). Determination of spore growth was done by using the MPN method. *B. subtilis* spores (an environmental isolate kindly provided by the State Office for Agriculture, Food Safety and Fishery of Mecklenburg-Western Pomerania, Rostock, Germany) and *M. senegalense* (Reference strain DSM 110653) were prepared as in Schinköthe et al. (5), and the plate counting method was used for determination of CFU per gram.

#### Environmental monitoring, disinfection efficacy, and statistical analysis

The efficacy of vaporized 35% H_2_O_2_ disinfection cycles (each *n* = 3) was assessed by calculating the log_10_ reductions by subtracting the logarithmic values of TCID_50_/mL, CFU/mL, or MPN/mL of each decontaminated germ carrier from the untreated (dried) positive controls (Tables S1 to S3). Means with standard deviation were calculated from the logarithmic values of three independent experiments. Measured values of relative humidity (%), temperature (°C), and H_2_O_2_ (ppm) were exported from the vendor software (Testo Comfort Basic, v 5.19, Testo, Germany), means were calculated and graphed as X-Y-diagrams.

In disinfection cycle one, the mean log_10_ reductions for each microorganism tested at locations 1–12 were further analyzed. Testing for normal distribution by the Shapiro-Wilk test returned a negative result, and therefore, a non-parametric univariate analysis of variance (Kruskal-Wallis test) with Dunn’s post hoc multiple comparisons test was performed.

To test the influence of relative humidity on the log_10_ reductions during the disinfection phase, all RH data points were extracted and matched with the mean RH calculated for locations 2, 6, 10, 14, and 22 for each of the three runs separately. The 15 RH values were used as the independent variable for the regression analysis, while the log_10_ reductions of tested microorganisms from all three runs were used as the dependent variable. A simple linear regression was performed with calculation of the *r*² and *P* values, and the results were plotted as an X-Y-diagram with a residual plot. We further computed Pearson’s correlation coefficient with respective *P* values. A *P* value of <0.05 was the accepted threshold for statistical significance.

To assess the effects of soil load and storage duration (30% RH, 22°C) on microbial viability, we used a two-way ANOVA with Šídák’s multiple-comparisons test (*α* = 0.05). Data are shown as mean ± SD from three technical replicates per time point. All statistical analyses, figures for RH and T, and heat maps were created with Prism version 11 (GraphPad Software, USA).

## SUPPLEMENTAL MATERIAL

Fig. S1 aem.01269-26-s0001.tif Infrared image, residual plot, chemical indicators, and germ carriers.

Supplemental legend aem.01269-26-s0002.docx Legend for Fig. S1.

Supplemental tables aem.01269-26-s0003.docx Tables S1 to S3.

ASM does not own the copyrights to Supplemental Material that may be linked to, or accessed through, an article. The authors have granted ASM a non-exclusive, world-wide license to publish the Supplemental Material files. Please contact the corresponding author directly for reuse.
