# Supplementary material for: Development of a functional and disinfectable sewage vent high-efficiency particulate air filter system and its validation using vaporized hydrogen peroxide
Source: Appl Environ Microbiol. 2026 Sep 9;92(10):e01269-26. doi: 10.1128/aem.01269-26 (PMC13688952; doi:10.1128/aem.01269-26)
Supplement: Supplementary file 1 [file aem.01269-26-s0002.docx]

**Fig S1** **(A)** Infra-red image showing the temperature gradient at germ carrier locations 17 and 21. **(B)** Residual plot of the simple linear regression model shown in Fig. 2G. **(C)** Chemical indicators and CSC before exposure to vH_2_O_2_ (upper panel) and after treatment (lower panel) with signs of marked surface damage (arrow) and extensive condensation on the tank surfaces (arrowheads). **(D)** Self-coated MNV germ carriers without (upper panel) and with soil load (lower panel); only GCs with soil load show marked bubble formation/effervescence (arrows).
