# Supplementary material for: Development of a functional and disinfectable sewage vent high-efficiency particulate air filter system and its validation using vaporized hydrogen peroxide
Source: Appl Environ Microbiol. 2026 Sep 9;92(10):e01269-26. doi: 10.1128/aem.01269-26 (PMC13688952; doi:10.1128/aem.01269-26)
Supplement: Supplementary file 2 [file aem.01269-26-s0003.docx]

**Table S1.** Log_10_ reduction of infectivity as mean and standard deviation (SD) of exposed germ carriers at distinct locations after fumigation with hydrogen peroxide.

| **GC** | **VSV** | | **GS(CSC)** | | **MS** | | **BS** | | **MNV** | |
| --- | --- | --- | --- | --- | --- | --- | --- | --- | --- | --- |
|  | **mean** | **SD** | **mean** | **SD** | **mean** | **SD** | **mean** | **SD** | **mean** | **SD** |
| 1 | 6.41 | 0.95 | 6.00 | 0.00 | 5.63 | 0.53 | 3.11 | 1.47 | 2.00 | 0.33 |
| 2 | 6.74 | 0.39 | 6.00 | 0.00 | 4.92 | 0.37 | 2.91 | 0.84 | 0.78 | 0.19 |
| 3 | 6.74 | 0.39 | 6.00 | 0.00 | 5.61 | 0.65 | 3.55 | 1.10 | 1.55 | 0.51 |
| 4 | 6.52 | 0.75 | 6.00 | 0.00 | 5.33 | 0.33 | 3.10 | 1.28 | 1.33 | 0.58 |
| 5 | 6.74 | 0.39 | 6.00 | 0.00 | 5.64 | 0.40 | 3.58 | 1.23 | 2.00 | 1.20 |
| 6 | 6.74 | 0.39 | 6.00 | 0.00 | 4.98 | 0.52 | 2.94 | 1.54 | 0.78 | 0.39 |
| 7 | 6.74 | 0.39 | 6.00 | 0.00 | 6.38 | 0.49 | 4.26 | 0.50 | 4.89 | 2.22 |
| 8 | 6.74 | 0.39 | 6.00 | 0.00 | 5.12 | 0.59 | 3.37 | 1.21 | 1.11 | 0.19 |
| 9 | 6.74 | 0.39 | 6.00 | 0.00 | 5.85 | 0.26 | 3.53 | 1.58 | 2.22 | 0.70 |
| 10 | 6.52 | 0.75 | 6.00 | 0.00 | 5.13 | 0.42 | 2.70 | 1.70 | 1.33 | 0.88 |
| 11 | 6.63 | 0.57 | 6.00 | 0.00 | 5.79 | 0.46 | 4.23 | 0.75 | 2.89 | 1.07 |
| 12 | 6.63 | 0.57 | 6.00 | 0.00 | 4.69 | 0.70 | 2.70 | 0.92 | 1.00 | 0.67 |
| 13 | 6.74 | 0.39 | 6.00 | 0.00 | 6.68 | 0.78 | 6.34 | 0.52 | 5.44 | 0.69 |
| 14 | 6.74 | 0.39 | 6.00 | 0.00 | 6.68 | 0.78 | 6.34 | 0.52 | 5.89 | 0.84 |
| 15 | 6.74 | 0.39 | 6.00 | 0.00 | 6.68 | 0.78 | 6.08 | 0.76 | 5.22 | 0.51 |
| 16 | 6.74 | 0.39 | 6.00 | 0.00 | 6.68 | 0.78 | 6.34 | 0.52 | 5.00 | 0.33 |
| 17 | 6.74 | 0.39 | 6.00 | 0.00 | 6.68 | 0.78 | 6.34 | 0.52 | 4.67 | 1.73 |
| 18 | 6.74 | 0.39 | 6.00 | 0.00 | 6.68 | 0.78 | 6.34 | 0.52 | 5.78 | 1.26 |
| 19 | 6.74 | 0.39 | 6.00 | 0.00 | 6.68 | 0.78 | 6.34 | 0.52 | 5.33 | 1.20 |
| 20 | 6.74 | 0.39 | 6.00 | 0.00 | 6.68 | 0.78 | 6.34 | 0.52 | 5.11 | 1.89 |
| 21 | 6.74 | 0.39 | 6.00 | 0.00 | 5.75 | 0.82 | 3.86 | 0.92 | 2.44 | 0.84 |
| 22 | 6.74 | 0.39 | 6.00 | 0.00 | 6.68 | 0.78 | 6.34 | 0.52 | 5.55 | 1.17 |
| 23 | 6.74 | 0.39 | 6.00 | 0.00 | 5.46 | 0.65 | 3.94 | 1.92 | 1.67 | 0.33 |
| 24 | 6.74 | 0.39 | 6.00 | 0.00 | 6.31 | 1.12 | 6.34 | 0.52 | 4.78 | 2.21 |

GC, germ carrier location; VSV, vesicular stomatitis virus; MNV, murine norovirus; MS, *Mycobacterium senegalense*; BS, *Bacillus subtilis*; green font, complete inactivation of GC.

**Table S2.** Log_10_ reduction of infectivity as mean and standard deviation (SD) of exposed germ carriers at distinct locations after fumigation with hydrogen peroxide.

| **GC** | **MNV** | | **GS** | |
| --- | --- | --- | --- | --- |
|  | **mean** | **SD** | **mean** | **SD** |
| 1 | 5.88 | 1.35 | 5.86 | 0.51 |
| *1** | *6.00* | *0.00* | *6.29* | *0.35* |
| 2 | 5.88 | 0.39 | 6.16 | 0.00 |
| 3 | 6.22 | 0.51 | 6.16 | 0.00 |
| 4 | 6.66 | 0.00 | 5.77 | 0.67 |
| *4** | *6.00* | *0.00* | *6.26* | *0.33* |
| 5 | 6.11 | 0.96 | 6.13 | 0.05 |
| *5** | *6.33* | *0.58* | *6.29* | *0.35* |
| 6 | 6.66 | 0.00 | 5.77 | 0.67 |
| 7 | 6.33 | 0.58 | 6.16 | 0.00 |
| 8 | 5.88 | 1.35 | 6.13 | 0.05 |
| *8** | *6.33* | *0.58* | *6.29* | *0.35* |
| 9 | 6.66 | 0.00 | 6.16 | 0.00 |
| 10 | 6.33 | 0.58 | 6.13 | 0.05 |
| 11 | 5.99 | 0.58 | 6.16 | 0.00 |
| *11** | *6.00* | *0.00* | *5.99* | *0.45* |
| 12 | 5.99 | 0.58 | 6.16 | 0.00 |
| 13 | 6.66 | 0.00 | 5.57 | 0.51 |
| 14 | 5.99 | 0.58 | 6.16 | 0.00 |
| 15 | 5.66 | 0.00 | 6.03 | 0.22 |
| 16 | 5.66 | 0.00 | 5.75 | 0.65 |
| 17 | 5.99 | 0.58 | 6.13 | 0.05 |
| 18 | 5.66 | 0.00 | 6.16 | 0.00 |
| *18** | *6.00* | *0.00* | *6.29* | *0.35* |

GC, germ carrier location; MNV; murine norovirus; GS, *Geobacillus stearothermophilus*; green font, complete inactivation of GC; *GC with organic soil load

**Table S3.** Mean titers of dried organism over time as decadic logarithm of tissue culture infectious dosis_50_/ml (viruses) or colony-forming units/gram (bacteria) with standard deviations stored at 30% relative humidity and 22°C.

| ***Viruses*** | | | | |
| --- | --- | --- | --- | --- |
| **Days** | **VSV** | **VSV soil-load** | **MNV** | **MNV soil-load** |
| **1** | 8.30 ± 0.20 | 8.72 ± 0.24 | 7.30 ± 0.20 | 7.88 ± 0.12 |
| **2** | 7.63 ± 0.31 | 7.97 ± 0.31 | 6.88 ± 0.12 | 7.47 ± 0.12 |
| **4** | 7.56 ± 0.20 | 7.88 ± 0.24 | 6.13 ± 0.12 | 6.97 ± 0.12 |
| **7** | 6.05 ± 0.35 | 7.30 ± 0.20 | 5.72 ± 0.12 | 6.97 ± 0.24 |
| **14** | 5.30 ± 0.20 | 6.72 ± 0.24 | 4.88 ± 0.31 | 6.63 ± 0.24 |
| **28** | 2.22 ± 0.24 | 4.97 ± 0.12 | 2.30 ± 0.20 | 5.38 ± 0.24 |
| ***Bacteria & Spores*** | | | | |
| **Days** | **MS** | **MS soil-load** | **BS** | **BS soil-load** |
| **1** | 8.19 ± 0.18 | 8.44 ± 0.29 | 8.12 ± 0.14 | 8.04 ± 0.07 |
| **2** | 8.11 ± 0.03 | 8.34 ± 0.20 | 8.20 ± 0.17 | 8.18 ± 0.05 |
| **4** | 7.03 ± 0.10 | 8.52 ± 0.08 | 8.22 ± 0.15 | 8.27 ± 0.12 |
| **7** | 4.69 ± 0.09 | 7.99 ± 0.10 | 8.12 ± 0.07 | 8.10 ± 0.23 |
| **14** | 4.16 ± 0.07 | 5.86 ± 0.51 | 7.99 ± 0.13 | 7.94 ± 0.30 |
| **28** | 2.85 ± 0.29 | 3.00 ± 0.00 | 8.15 ± 0.02 | 8.11 ± 0.16 |

VSV, vesicular stomatitis virus; MNV, murine norovirus; MS, *Mycobacterium senegalense*; BS, *Bacillus subtilis*.
